# Comparison of Sexual Behavior and HIV Risk between Two HIV-1 Serodiscordant Couple Cohorts: The CHAVI 002 Study

**DOI:** 10.1371/journal.pone.0037727

**Published:** 2012-05-22

**Authors:** Adam J. Ritchie, Kristin Kuldanek, Zoe Moodie, Z. Maggie Wang, Julie Fox, Rebecca N. Nsubuga, Kenneth Legg, Esther F. Birabwa, Pontiano Kaleebu, Andrew J. McMichael, Christine Watera, Nilu Goonetilleke, Sarah Fidler

**Affiliations:** 1 Weatherall Institute of Molecular Medicine, University of Oxford, Oxford, United Kingdom; 2 HIV Clinical Trials Centre, Imperial College, St Mary’s Hospital, London, United Kingdom; 3 Statistical Center for HIV/AIDS Research and Prevention, Fred Hutchinson Cancer Research Center, Seattle, Washington, United States of America; 4 Department of HIV, Faculty of Medicine, King's College, Guy and St Thomas’ Hospital, London, United Kingdom; 5 MRC/UVRI Uganda Research Unit on AIDS, Entebbe, Uganda; University of Alabama at Birmingham, United States of America

## Abstract

**Background:**

The CHAVI002 study was designed to characterize immune responses, particularly HIV-specific T-cell responses, amongst 2 cohorts of HIV-exposed seronegative (HESN) individuals. The absence of a clear definition of HESNs has impaired comparison of research within and between such cohorts. This report describes two distinct HESN cohorts and attempts to quantify HIV exposure using a ‘HIV risk index’ (RI) model.

**Methods:**

HIV serodiscordant couples (UK; 24, Uganda; 72) and HIV unexposed seronegative (HUSN) controls (UK; 14, Uganda; 26 couples, 3 individuals) completed sexual behavior questionnaires every 3 months over a 9 month period. The two cohorts were heterogeneous, with most HESNs in the UK men who have sex with men (MSM), while all HESNs in Uganda were in heterosexual relationships. Concordance of responses between partners was determined. Each participant’s sexual behavior score (SBS) was estimated based on the number and type of unprotected sex acts carried out in defined time periods. Independent HIV acquisition risk factors (partner plasma viral load, STIs, male circumcision, pregnancy) were integrated with the SBS, generating a RI for each HESN.

**Results:**

96 HIV serodiscordant couples completed 929 SBQs. SBSs remained relatively stable amongst the UK cohort, whilst decreasing from Visit 1 to 2 in the Ugandan cohort. Compared to the Ugandan cohort, SBSs and RIs in the UK cohort were lower at visit 1, and generally higher at later visits. Differences between the cohorts, with lower rates of ART use in Uganda and higher risk per-act sex in the UK, had major impacts on the SBSs and RIs of each cohort. There was one HIV transmission event in the UK cohort.

**Conclusions:**

Employment of a risk quantification model facilitated quantification and comparison of HIV acquisition risk across two disparate HIV serodiscordant couple cohorts.

## Introduction

The overall risk of sexual HIV transmission reflects the frequency and nature of exposure to virus with different sex acts confer varying risks of transmission [1]–[10]. In addition the susceptibility of an HIV uninfected individual, and the infectiousness of their HIV seropositive partner contribute to transmission risk [2]. The concept that certain rare individuals remain HIV uninfected despite repeated sexual exposure to an HIV infected partner has long been described [11]–[18]. Whilst HIV exposed seronegative (HESN) individuals have provided extensive information on the biology of HIV transmission, the relative and specific contributions of host and viral factors underlying HIV ‘resistance’ remain unclear. The absence of a consistent definition of HESNs and inclusion of well-defined control groups has complicated comparisons both between and within studies [19]. Unanswered questions remain about the frequency, type of sex, and duration of exposure required to define a HESNs phenotype. Many studies of sexual behavior have focused on either men who have sex with men (MSM) or heterosexual populations, and it is difficult to extrapolate or compare data from one to the other due to differences in risk associated with, for example, vaginal or anal intercourse [20].

Robust methodologies to quantify risk will facilitate a clear definition of truly HESN cohorts. The primary goal of the CHAVI002 study was to analyse immune parameters associated with the HESN phenotype [21]. We report here on the secondary goal of this study, which was to apply a detailed sexual behavior questionnaire (SBQ) facilitating comparisons of reported behaviors between HIV serodiscordant couples and HIV unexposed seronegative (HUSN) controls in the context of detection of HIV-specific immune responses. This SBQ, which details the frequency and type of unprotected sex acts by couples, allows the quantification of behavior based HIV-1 infection risk based on population-level transmission data from serodiscordant couple studies [20]. In the current study the SBQ was applied to two diverse serodiscordant couple cohorts; a mostly MSM cohort based in the United Kingdom, and a heterosexual cohort based in Uganda. Sexual behavior analysis was coupled with clinical data to build an HIV transmission risk index (RI) estimate for HESN participants based on published literature [20]. Although the SBS and RI generated as part of the CHAVI002 study was not found to correlate with the detection of HIV-1 specific T cell responses [21], analyses in the context of other immune parameters are ongoing. The ability to improve the stratification of HESN within cohorts, and compare levels of exposure between cohorts, will allow an improved understanding of what immunological factors play the most significant roles in resistance to HIV-1 infection, and if such factors vary depending on the level and route of exposure.

## Methods

### Population Definitions

Two HIV serodiscordant couple cohorts were enrolled using the same definition at: Imperial College NHS Trust London, UK, (24 serodiscordant, 14 HUSN couples) and MRC/UVRI, Entebbe, Uganda (72 serodiscordant, 26 HUSN couples plus 3 HUSN individuals). Potential participants were recruited through sexual health clinics, local advertisements, and word of mouth. The study protocol was approved by Division of AIDS, National Institutes of Health and local ethics committees. All individuals enrolled gave written informed consent. Condoms and risk reduction counselling were given to all participants at each study visit; continued unsafe sex practises were discouraged. Injection drug use was an exclusion criterion whilst antiretroviral therapy (ART), pregnancy, and breastfeeding were not. All participants were ≥18 years old. HESN inclusion criteria were developed to ensure both minimal levels and periods of exposure were met, while HUSN inclusion criteria were developed to ensure individuals were sexually active but at minimal risk of having been exposed to HIV-1 on any level for at least 1 year.

### Inclusion Criteria

### HIV Serodiscordant Couples

- First sex act within the couple occurred ≥10 months prior to screening
- ≥25 occasions of unprotected vaginal, anal, and/or oral sex within the preceding 12 months
- The HIV infected partner was diagnosed ≥12 months prior to screening
- HESN partner HIV-1 antibody and virus (NAT or p24 antigen) negative

### Control HUSN Couples*

- Both partners HIV-1 antibody and virus (NAT or p24 antigen) negative
- Free from sexually transmitted infections (STIs) at screening
- Monogamous sexual relationship for ≥12 months
* In the Ugandan cohort, it was permitted for 1 individual of a confirmed HUSN couple to enrol without their partner. Although no data from such individuals were included in the concordance, SBS, or RI analyses, samples were made available for cellular studies.

Couples were followed for 4 study visits over 9 months. Data collection, management and monitoring were undertaken by the Statistical Center for HIV/AIDS Research & Prevention (SCHARP).

### HIV Testing

Determine® HIV-1/2 point-of-care 3^rd^ generation immunoassay, and laboratory HIV NAAT assay (Micropathology Ltd., In-house nested NAAT) were performed on all HIV uninfected participants at each visit. Test standardisation was undertaken using quality assurance panels (UKNEQAS in the UK and UKNEQAS, CAP and VQA in Uganda). HIV plasma virus load (pVL) (UK; Siemens Versant HIV-1 RNA 3.0 Assay, LOD; 50 copies/ml Uganda; Roche Amplicor v1.5 HIV test, LOD; 400 copies/ml), CD4 T-cell count (measured using FACS analysis), and viral genotype were performed in the HIV+ participants.

### STI Screening

Genital examinations and STI screens were offered at each study visit. Any STI diagnosed was treated according to local guidelines. Gonorrhoea and chlamydia PCR testing were performed using the BD ProbeTec ET Strand Displacement Amplification test on urine in men and endocervical sampling in women. ELISA testing for syphilis, HSV-2 (UK only), and hepatitis A, B, and C (Siemens ADVIA Centaur immunoassay) were performed. Genital ulceration was diagnosed using ELISA for syphilis and viral PCR for herpes.

### Sexual Behavior Questionnaires (SBQs)

Individual, confidential, structured, paper SBQs were administered by one research nurse in the UK and 3 counsellors in Uganda. They were collected at every visit, recording the total number of protected and unprotected sex acts for the: past 3 months, past 7 days, and most sexually active week over the preceding 3 months. These 3 time-frames were included for 2 reasons; firstly to allow an analysis of the concordance of responses between the time windows, and secondly different time periods were more appropriate for correlation with various immune parameters. Six key sex acts, involving no condoms and ejaculation, were further evaluated in this study: i) receptive anal intercourse (RAI), ii) insertive anal intercourse (IAI), iii) insertive vaginal intercourse (IVI), iv) receptive vaginal intercourse (RVI), v) receiving oral intercourse (R-OI), and vi) giving oral intercourse (G-OI).

### Concordance of SBQ Responses between Study Partners

Concordance in SBQ responses was defined arbitrarily as a<3 fold difference in frequencies reported within a couple, or if one partner gave an answer of 0, where the other answered <3. Lack of concordance in responses for 1 or more sex acts at a particular visit excluded analysis of SBQ data from that couple for that visit.

### Estimation of Sexual Behavior Scores (SBSs)

Where concordance criteria were met, individual SBSs were estimated over three time frames. SBS represents a composite of frequency (n) of each unprotected (U) high risk sex act; URAI, UIAI, UIVI, and URVI and is the sum of the number of times each act was performed by the individual in the preceding time frame, multiplied by the estimated per contact risk for the given act, based on literature from HIV serodiscordant couple studies [20]. Even though there is no per contact risk for the HUSN and HIV+ participants, as their partners were HIV negative, their SBSs were still calculated. This was used as a weighted marker for the sex acts carried out, and is useful as a baseline measure of how sexual behaviors change over time in the absence of a perceived risk of infection, and how risk is distributed amongst serodiscordant MSM couples.



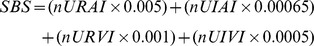
.

### HIV Risk Index

An overall risk index (RI) was then estimated for each HESN participant at each visit based on the previous 3 month period. While in the current study the RI is not a reflection of the probability of an HIV-1 transmission event occurring, it does indicate the relative risk of transmission. The RI is the integration of the SBS with known independent risk factors; partner HIV-pVL, male circumcision status, pregnancy, and other STIs. This is achieved by multiplication of the SBS based on relative risks and odds ratios reported from HIV sero-discordant couple studies [2], [20]. Several potential risk factors were excluded from the current study due to a lack of any incidences being reported (infectious syphilis), all participants being the same for this factor (stage of HIV infection), or a lack of consistent collection of relevant data across study sites (HSV-2 serostatus, bacterial vaginosis). The odds ratios used to generate the RI are outlined in Table 1, and employed as outlined in the following formula.

**Table 1:** The risk factors and odds ratios used to generate the risk index (RI) in the current study.

Factor	HESN susceptibility	HIV+ infectiousness
Viral load (Log_10_)		
0–3.49		1
3.50–4.16		5.8
4.17–4.88		6.91
>4.88		11.87
Male circumcised	0.47	1
GUD	2.58	2.04
Pregnancy	2.16	1



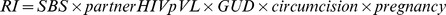
.

### Statistical Analyses

Two-sided Mann-Whitney tests with significance level of 0.05 were used to compare continuous variables between the two study cohorts (e.g., CD4 counts, SBS). Two-sided Fisher’s exact tests with significance level of 0.05 were used to compare response rates (e.g., circumcision rates, ART use rates, STI rates, and the proportion of MSMs). No adjustments were made for multiple testing.

## Results

### Characteristics of Study Participants

Table 2 summarises the characteristics of the two cohorts recruited. The HESN and HUSN groups are highly heterogeneous. In the UK cohort, HESN consist of 96% (23/24) MSMs while HUSN are 36% (10/28) MSMs (p<0.0001), 32% (9/28) heterosexual men, and 32% women. Ugandan couples reported longer relationships (median 64, 74 months) for serodiscordant and HUSN couples respectively than in the UK (median 23, 39 months). A greater proportion of HIV+ individuals in the UK were on ART at visit 1 (70.8% UK, 34.7% Uganda, p = 0.004). Median CD4+ T cell counts were higher in the UK (560 cells/cc) than Ugandan cohort (234 cells/cc) at enrolment (p<0.0001). HIV-pVLs were much lower in the UK (median = LOD = <50 copies/cc) than in Uganda (median = 7,290 copies/cc). There was a lower rate of male circumcision amongst HESNs in the UK cohort (8%) compared to those in Uganda (47%) but this difference was not statistically significant (p = 0.22). The differences in circumcision rates for the HIV+ and HUSN groups were also not significant though rates were lower in the UK than in Uganda (9% vs 26% with p = 0.11 and 16% vs 38% with p = 0.12 respectively). There was a significantly higher prevalence of STIs observed in the Ugandan cohort, particularly in the HUSN group, where only 1/24 (4%) of UK HUSNs but 23/55 (42%) of Uganda HUSNs had any non-HIV STIs at any study visit (p = 0<0.0005). This difference is due to higher rates of bacterial vaginosis and candidiasis, in Uganda neither of which were reported in the UK cohort. Of the 23 HUSNs with episodes of STIs in Uganda, 18 reported bacterial vaginosis and/or candidiasis and no other STIs.

**Table 2:** Summary of Study Group Characteristics for UK and Uganda.

	UK				Uganda		
	HESN (n = 24)	HIV+ (n = 24)	HUSN (n = 28)		HESN (n = 72)	HIV+ (n = 72)	HUSN (n = 55)
**Age in years; median (IQR)** a	35 (30.5–47)	37 (29–43)	29.5 (27–33.5)		30 (26.5–40.5)	34.5 (28–40.5)	32 (26–37)
**Males**	24 (100%)	23 (96%)	19 (68%)		34 (47%)	38 (53%)	29 (53%)
**Males circumcised**	2/24 (8%)	2/23 (9%)	3/19 (16%)		16/34 (47%)	10/38 (26%)	11/29 (38%)
**MSM (% of all)** b	23 (96%)	23 (96%)	10 (36%)		0 (0%)	0 (0%)	0 (0%)
**White race**	24 (100%)	22 (92%)	24(86%)		0 (0%)	0 (0%)	0 (0%)
**Black African/Carribean**	0 (0%)	0 (0%)	2 (7%)		72 (100%)	72 (100%)	55 (100%)
**Months in relationship; median (IQR)**	23 (15–54)	20 (15–41)	39 (33–55)		64 (37–149)	66 (36–151)	74 (38–112)
**Prevalence of active STIs; any visit** c	2/24 (8%)	4/24 (17%)	1/24 (4%)		14/72 (19%)	11/72 (15%)	23/55 (42%)
**ART use at Visit 1 among HIV+**	N/A	17 (70.8%)	N/A		N/A	25 (34.7%)	N/A
**pVL for all HIV+ at Visit 1 median (range)**	N/A	n = 23^c^<50^e^(<50–340 581)	N/A		N/A	n = 71^d^7,290 (<400^e^-2 830 000)	N/A
**pVL for HIV+ on ART at Visit 1 median (range)**	N/A	n = 16<50 (<50–170)	N/A		N/A	n = 24<400 (<400–51 400)	N/A
**pVL for HIV+ not on ART at Visit 1; median (range)**	N/A	n = 7 14 054 (<50–340 581)	N/A		N/A	n = 47 21 900 (<400-2 830 000)	N/A
**CD4+ count for all HIV+ at Visit 1; median (range)**	N/A	n = 23^f^560 (10–1080)	N/A		N/A	n = 72 233.5 (49–735)	N/A
**CD4+ counts for HIV+ on ART at Visit 1; median (range)**	N/A	n = 16 540 (230–1080)	N/A		N/A	n = 25 265 (77–641)	N/A
**CD4+ counts for HIV+ not on ART at visit 1; median (range)**	N/A	n = 7 690 (10–789)	N/A		N/A	n = 47 229 (49–735)	N/A

a IQR; Interquartile range.

b No participants self-identified as bisexual, therefore those not identified as MSM are heterosexual.

c Participants positive for Chlamydia, Gonorrhoea, GUD, Bacterial Vaginosis, Candidiasis, and/or Syphilis at any study visit, and/or reporting incidences of genital lesions/discharge/rash/itching in the 3 months preceding any study visit.

d No pVL data available for one UK and one Ugandan HIV+ participant at visit 1.

e Limit of detection 50 copies/ml in UK and 400 copies/ml in Uganda samples.

f No CD4+ count data for one UK HIV+ participant at visit 1.

### SBQ Responses

A summary of the key reported SBQ responses for visit 1 is presented in Table 3. There was a wide range of reported sexual practices amongst all cohorts enrolled. Of note, there was no anal and limited oral intercourse reported, irrespective of risk group, in the Ugandan cohort.

**Table 3:** Data from visit 1 summarising key sexual behavior acts (with ejaculation and no condom use) reported within the past 3 months and the proportion of couples who were concordant in their responses across all sex acts.

	UK				Uganda		
	HESN n = 24	HIV+ n = 24	HUSN n = 28		HESN n = 72	HIV+ n = 72	HUSN n = 55
Insertive anal intercourse median (range)	6 (0–40)	0 (0–3)	3 (0–12)		NR^a^	NR	NR
Receptive anal intercourse median (range)	0 (0–2)	4 (0–50)	3.5 (0–38)		NR	NR	NR
Vaginal intercourse median (range)	3^b^	12^b^	19 (0–36)		12 (0–150)	12 (0–524)	30 (0–48)
Received oral sex median (range)	2 (0–24)	0 (0–50)	3.5 (0–30)		1 (0–14)	9 (0–24)	4 (0–42)
Gave oral sex median (range)	0 (0–36)	1 (0–50)	4 (0–30)		2 (0–18)	1 (0–3)	2 (0–42)
Concordance of responses	HESN/HIV+24 couples		HUSN 14 couples		HESN/HIV+72 couples		HUSN 25 couples
Past 7 days							
With oral	20/20 (100%)		13/13 (100%)		28/32 (88%)		22/22 (100%)
Without oral	20/20 (100%)		13/13 (100%)		29/30 (97%)		22/22 (100%)
Past 3 months							
With oral	12/24 (50%)		8/14 (57%)		55/69 (80%)		19/25 (76%)
Without oral	19/24 (79%)		11/14 (79%)		58/69 (84%)		20/25 (80%)
Most active week							
With oral	20/24 (83%)		13/13 (100%)		64/70 (91%)		25/25 (100%)
Without oral	20/24 (83%)		13/13 (100%)		64/70 (91%)		25/25 (100%)

a NR; none reported.

b Single data point only.

### Concordance of SBQ

Concordance of SBQ answers reflects the agreement in partners’ responses for each specific time period; results for visit 1 are provided in Table 3. Concordance was higher when oral sex questions were excluded. Concordance generally improved at later visits, with Visit 4 concordance reaching 93% (14/15) for UK serodiscordant couples’ responses for the past 3 months excluding oral sex questions (11/11 = 100% for HUSN couples) and 93% (43/46) for Ugandan serodiscordant couples (16/19 = 84% for HUSN couples).

### Comparisons of SBS for Projected and Observed Last 3 Month Data

To evaluate the agreement in reported behavior over different time frames, we compared the SBS based on the projected 3 month data (last 7 days multiplied by 13) to the SBS based on the reported 3 month data for HESN individuals with concordant data for both time frames. Overall, there is good agreement between the observed and projected 3 month SBSs (data not shown). However, the most significant observation was that for individuals where the number of sex acts in the past 7 days is low or zero, the projected 3 month SBS may not accurately reflect the 3 month exposure. In view of these discrepancies on an individual level, RI estimates were generated based on the observed 3 month SBS.

### Sexual Behavior Scores Over Preceding 3 Month Period

**Figure 1:**
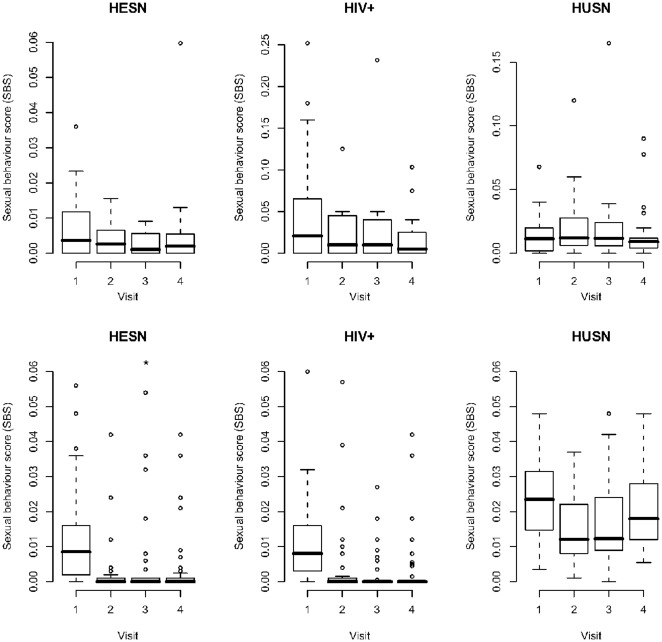
Sexual behavior scores (SBSs) across visits for study groups in the UK^1^ (top row) and Uganda (bottom row) cohorts. Boxplots of SBSs of concordant individuals are shown, where the midline and box represent the median ± the 25th and 75th percentiles; whiskers extend to the extreme data points that are ≤1.5 times the interquartile range. Extreme outliers are indicated by circles. ^1^Note that the scales of the y-axis for UK HIV+ and HUSN are much larger than those of the other groups. *One UK HESN with a very large SBS at Visit 3 (0.126) is not shown in order to maintain a comparable scale over plots where possible.

In the UK cohort, median SBSs for HESNs at Visit 1 was 0.003575 (range 0–0.036), 0.01130 (range 0–0.0678) for HUSNs and 0.02065 (range 0–0.25195) for HIV+ individuals. The highest SBS over all visits was 0.126 for HESNs (Visit 3), 0.165 for HUSNs (Visit 3), and 0.25195 for HIV+s (Visit 1). For both cohorts, visit 1 is the most relevant time point here, because it represents the baseline sexual behavior not yet influenced by safe sex counselling. There was some indication of a lowering of SBS over time in both the HESN and HIV+ groups, while HUSN SBSs remained more constant over time (Figure 1). Interpretation of a numerical value given for SBS is demonstrated using a representative individual with an initial high SBS (e.g. 0.25195) reflected 53 sex acts over the preceding 3 month period. For the same participant at another visit a SBS of 0.2315 represented 55 sex acts. Despite a slightly increased number of reported unprotected sex acts at this visit, the lower SBS reflects an increasing proportion of insertive rather than receptive sex acts.

In the Ugandan cohort, median SBS for HESN at Visit 1 was 0.00850 (range 0–0.075), 0.02350 (range 0–0.048) for HUSNs, and 0.00800 (range 0–0.0600) for the HIV+ individuals. In all study groups, there was a marked decrease in the SBS between visit 1 and visit 2 (Figure 1). A representative high SBS (0.076) was a female HESN reporting 76 unprotected vaginal sex acts over the preceding 3 month period.

There was a statistically significant difference (Mann Whitney tests) in SBS between the UK and Ugandan cohort for HESNs at Visits 2, 3, 4 (p = 0.044, 0.020, 0.023 respectively), for HUSNs at Visits 1 and 4 (p = 0.007 and 0.003), and for HIV+s at visits 2, 3, 4 (p = 0.038, 0.0002, 0.004), with the UK cohorts having higher SBSs across the HESN and HIV+ groups, and lower SBSs amongst the HUSN group.

### HESN Risk Index

Median RIs at visits 1, 2, 3, and 4 in the UK were 0.00663, 0.00305, 0.00195, and 0.00221, ranging over all visits from 0–0.126. Median RI at visit 1 in Uganda was 0.01949 and 0 at all later visits, ranging over all visits from 0–0.66472. The addition of HSV sero-status for the UK participants increased the RIs of HSV-2 seropositive participants (data not shown) but was not included in these analyses due to a lack of data from the Uganda cohort.

**Figure 2:**
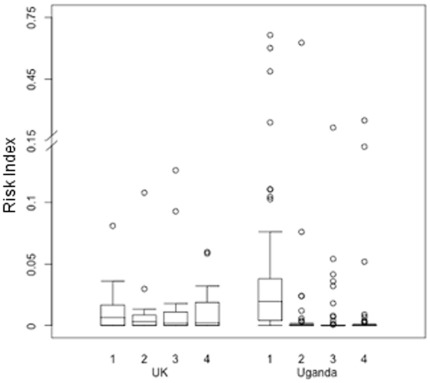
Risk indexes (RIs) for HESN participants in the UK and Ugandan cohorts over 4 visits. RIs were generated based on reported sexual behaviors for the last 3 months (if concordant with answers from the HIV+ study partner), partner HIV-1 pVL, concurrent STIs, male circumcision status, and pregnancy. Boxplots of RI are shown, where the midline and box represent the median ± the 25th and 75th percentiles; whiskers extend to the extreme data points that are ≤1.5 times the interquartile range. Extreme outliers are indicated by circles.

Figure 2 illustrates the overall RIs for all HESN participants for both Ugandan and UK participants over time in study. The relatively increase in RIs compared to SBSs seen at visit 1 in the Ugandan cohort compared with the UK demonstrates the impact of HIV+ partners’ pVL and pregnancy. After visit 1 it can be seen that the RI of UK HESN is higher than the Ugandan HESNs, due to a decrease in Ugandan SBSs.

### RI of Individual UK HESN Who Seroconverted

One HESN seroconverted between visits 1 and 2, and matched viral genotyping confirmed transmission from their study partner. For this participant the estimated SBS for visit 1 was 0.00065, and their partner’s pVL was 18 751 copies HIV RNA/ml, giving a RI of 0.00449.

### Discussion

Whilst the development of risk evaluation models remains controversial [22], [23] the absence of an internationally agreed definition of HESN necessitated a pragmatic definition of the two cohorts for this study. We have shown that a similarly structured SBQ can be used in conjunction with clinical data to allow comparison of HIV-1 exposure between two very different HESN cohorts; a primarily MSM cohort from the UK and an entirely heterosexual cohort from Uganda. Rigorous enrolment criteria were used to maximise the levels of exposure defining HESNs in this study, Analysis of the SBS and RI describes a wide heterogeneity in the levels of relative HIV-1 exposure for individual HESN participants, both between and within cohorts. This was largely influenced by differences in sexual behaviors (including the type and frequency of sex acts), pVLs and ART use in seropositive partners. This heterogeneity underpins much of the controversy of immunological data in the HESN field [11]–[18].

The main goal of the CHAVI002 study was to characterise HIV-specific immune responses amongst HESNs and HUSNs [21]. For this reason enrolment of appropriate matched for sexual practices, unexposed control populations defined by a SBS was critical to allow definitive comparison in immunological outcome measures. However, despite these important goals, the enrolment of suitable HUSN couples was challenging, necessitating protocol amendments. Difficulties in enrolling monogamous MSM HUSN couples were encountered in the UK. In Uganda although many HUSN couples expressed an interest in the study, both partners did not always agree to participate.

Sexual behavior reported through a SBQ is often confounded by bias introduced by participant’s wish to please, recall bias, and interviewer bias [24]–[26]. The use of self-completed or computer-led SBQs (e.g. CASI) can circumvent some of these specific issues but have their own potential to introduce bias [27], [28]. The absence of reported anal intercourse amongst the Ugandan participants across all 3 risk groups was unexpected [10], [29]–[32], as other African studies populations reported 1 in 50 heterosexual sex acts involved anal sex [31]. This may be linked to discomfort about the practice among interviewers and participants and the belief that anal intercourse represents homosexual practice. In our analyses it is not possible to account for bias in sexual behavior reporting. The data from visit 1 represents the most accurate representation of pre-study sexual behavior, and subsequently practices altered, mostly reducing risk. We would recommend removing questions relating to ‘the week of most activity’ as this time frame was poorly defined, and often misinterpreted by couples leading to discrepant responses. Similarly poor concordance between partners in reported oral sex act frequency led to its removal in calculating the SBS and RI. This may reflect difficulties in interpreting relevant questions, as oral sex may form an integral part of the main penetrative sex act rather than a separate act itself. The recent linkage of reported oral exposure with systemic immunological outcomes [18] suggests that oral sex practices are valuable in evaluating overall risk behaviors.

The rates of STIs may be underestimated in the Ugandan cohort as, although STI screens were offered to all participants only 33% of participating individuals consented to full STI screening at every study visit. Many diagnoses were based on self-reported symptoms, especially of genital ulcer disease (GUD) and for this reason GUD was excluded as a factor when estimating the overall RIs for all HESNs in this study. If such a tool were to be utilised in the future, we would recommend the incorporation of GUD screening. HSV-2 serology was performed for UK HESNs only, hence the Uganda RIs could not incorporate HSV-2 serostatus information and thus this factor was not considered in this study but could be in future ones. There are several other factors that have not been included in the RI in this study such as genetic [13], immunological [11]–[18], and viral characteristics [2], [10], [33], [34], and sex outside the primary relationship. Overall, the exclusion of these factors means that the RIs reported may be an underestimate of the true risk of HIV-1 transmission.

The decision to focus SBS and RI estimates and comparisons on sex acts reported for the previous 3 month period was based upon the greater biological applicability of this data to adaptive immune responses and thus the primary goal of the CHAVI 002 study; analysis of T-cell responses [21]. Analyses based on sexual exposure over the preceding 7 days may be more relevant to studies examining innate and/or mucosal immunity [35].

SBS estimates from visit 1 most accurately represent sexual exposure prior to enrolment into the study. The observed decrease in SBSs over time amongst the Ugandan HESN may result from improved safe sex education, provision of free condoms and STI screening. The relative stability in SBSs amongst the HESN MSM population in the UK could reflect a longer history of safe sex awareness in this group, meaning the unsafe acts occurring prior to visit 1 were not due to ignorance, and reinforced by repeated HIV negative test results. This is supported by the differences in the UK HESN and HIV+ SBSs, with the later group having approximately 5 fold higher scores, suggesting behavioral risk stratification being employed by these serodiscordant couples. However, the one HIV transmission observed during this study illustrates the danger of ambivalence to unsafe sexual practices within high risk populations. Appropriate individualised safe sex counselling remains critical for each HESN and is key to delivering lasting behavior changes. The apparent resistance to such messages in the UK MSM cohort indicates that in the future more innovative methods for delivery of safe sex messages should be implemented [36].

The development of the RI model to evaluate relative risk of HIV acquisition in the context of HIV serodiscordant couples has been previously outlined in detail [20]. The quality of all models is dependent on the validity of the underlying assumptions and accuracy of the supporting data used to develop it. The absolute value for each individual HESN RI represents their overall modelled risk of HIV acquisition for a specific time period. For this RI model the supporting evidence was derived from published clinical data from HIV serodiscordant couple studies. More recent estimates of transmission risk suggest a 20-fold greater risk for anal than vaginal intercourse [10], which is greater than the difference in the RI model used here, and a strong decrease in transmission risk related to early ART intervention [37] suggesting that RI models should be subject to ongoing review as more informative data becomes available. Issues with answers to questions relating to oral sex, the lack of information gathered on sex outside the primary relationship, and the exclusion of other factors that can influence risk, such as HSV-2 sero-status, could have led to an underestimation of RIs. For the purposes of this paper, the RIs were mainly of interest for cross-cohort and longitudinal comparisons rather than an absolute measure of risk. However, for comparisons across cohorts one must be aware of the potential for differences in reporting sexual behavior; it is not possible to account for this. Without validation against empirical data, one cannot know whether the proposed risk index is reasonable, either in absolute terms (making inferences about an individual’s probability of acquiring HIV) or in relative terms (comparing acquisition risks in heterosexual men, heterosexual women and MSM). Future work to validate this model is proposed, from transmissions recorded through couple studies [37]. This study highlights that a SBS/RI approach can be used to quantify relative risk in HESN cohorts, and that such risk can be highly heterogeneous even when strict enrolment criteria are used.
